# A community-based mobile clinic model delivering PrEP for HIV prevention to adolescent girls and young women in Cape Town, South Africa

**DOI:** 10.1186/s12913-021-06920-4

**Published:** 2021-08-28

**Authors:** Elzette Rousseau, Linda-Gail Bekker, Robin F. Julies, Connie Celum, Jennifer Morton, Rachel Johnson, Jared M. Baeten, Gabrielle O’Malley

**Affiliations:** 1 Desmond Tutu Health Foundation, University of Cape Town, Cape Town, South Africa; 2 Department of Global Health, University of Washington, Seattle, USA

**Keywords:** HIV prevention, Pre-exposure prophylaxis (PrEP), Adolescent girls and young women (AGYW), Community mobile health clinic (CMHC), PrEP delivery models, Sexual reproductive health services (SRHS)

## Abstract

**Background:**

Daily doses of pre-exposure prophylaxis (PrEP) can reduce the risk of acquiring HIV by more than 95 %. In sub-Saharan Africa, adolescent girls and young women (AGYW) are at disproportionately high risk of acquiring HIV, accounting for 25 % of new infections. There are limited data available on implementation approaches to effectively reach and deliver PrEP to AGYW in high HIV burden communities.

**Methods:**

We explored the feasibility and acceptability of providing PrEP to AGYW (aged 16–25 years) via a community-based mobile health clinic (CMHC) known as the Tutu Teen Truck (TTT) in Cape Town, South Africa. The TTT integrated PrEP delivery into its provision of comprehensive sexual and reproductive health services (SRHS). We analyzed data from community meetings and in-depth interviews with 30 AGYW PrEP users to understand the benefits and challenges of PrEP delivery in this context.

**Results:**

A total of 585 young women started PrEP at the TTT between July 2017 – October 2019. During in-depth interviews a subset of 30 AGYW described the CMHC intervention for PrEP delivery as acceptable and accessible. The TTT provided services at times and in neighborhood locations where AGYW organically congregate, thus facilitating service access and generating peer demand for PrEP uptake. The community-based nature of the CMHC, in addition to its adolescent friendly health providers, fostered a trusting provider-community-client relationship and strengthened AGYW HIV prevention self-efficacy. The integration of PrEP and SRHS service delivery was highly valued by AGYW. While the TTT’s integration in the community facilitated acceptability of the PrEP delivery model, challenges faced by the broader community (community riots, violence and severe weather conditions) also at times interrupted PrEP delivery.

**Conclusions:**

PrEP delivery from a CMHC is feasible and acceptable to young women in South Africa. However, to effectively scale-up PrEP it will be necessary to develop diverse PrEP delivery locations and modalities to meet AGYW HIV prevention needs.

**Supplementary Information:**

The online version contains supplementary material available at 10.1186/s12913-021-06920-4.

## Background

Despite global acceleration of the HIV/AIDS response since 2010, 1.7 million new HIV infections occurred in 2018 [1]. In sub-Saharan Africa, adolescent girls and young women (AGYW) aged 15–24 years are at disproportionately high risk of acquiring HIV, accounting for 25 % of new infections [2]. The benefit of pre-exposure prophylaxis (PrEP) for HIV prevention has been well-established. Daily doses of oral PrEP (Emtricitabine/Tenofovir Disoproxil Fumarate) have been shown to reduce the risk of acquiring HIV by more than 95 % when adherence is high [3–5]. PrEP effectiveness at the population level will depend on how well AGYW can access, adhere, and persist with PrEP [6, 7]. There are limited data available on implementation approaches to efficiently reach and deliver PrEP to women in high HIV burden communities, particularly adolescent girls and young women.

Adolescence into early adulthood is a period of significant physiological and psychological changes, and often the occurrence of sexual debut, making trustworthy and reliable sexual reproductive health services (SRHS) and information important [8–10]. Young women’s health-seeking behaviours are shaped by their life contexts and relationship with the public healthcare system [11]. AGYW in South Africa have described barriers to SRHS access due to increased travel distance to health facilities, long waiting times at overcrowded facilities, negative healthcare provider attitudes about sexual activity among AGYW, negative social consequences resulting from a lack of privacy at SRHS delivery points, and limited availability of adolescent friendly health services [10–14]. The challenges of reaching AGYW and supporting their PrEP use have similarly been described from initial PrEP clinical trials [15–18]. Programs will have to overcome these barriers to successfully engage AGYW to utilize PrEP as an HIV prevention strategy.

Community-based mobile health clinics (CMHC) are an important model for delivering health services to vulnerable populations and impactful when providing differentiated, tailored and client-centered preventative health services [19–21]. In low- and middle-income countries, mobile health clinics have been deployed to provide a wide range of public health services; including cervical cancer screening in Thailand [22] and Brazil [23], maternal and child health care in Tanzania [24], primary health care in Namibia [25] and Malawi [26], mental health and diabetes screening and services in India [27, 28], and HIV screening and sexual and reproductive health services in South Africa [29, 30]. CMHC that provide comprehensive integrated SRHS may be especially attractive to AGYW, providing flexibility in location to increase accessibility for these populations in resource-limited settings.

This paper describes a novel approach to delivering PrEP as an integrated component of community-based comprehensive SRHS to AGYW via a mobile health clinic in Cape Town, South Africa.

## Methods

### Study context

POWER (Prevention Options for Women Evaluation Research) is a PrEP implementation science project testing scalable models of PrEP delivery for young women, ages 16–25, in South Africa and Kenya. PrEP delivery is being incorporated into established health service settings, including an adolescent friendly clinic (Johannesburg, South Africa), family planning clinics (Kisumu, Kenya) and a mobile clinic (Cape Town, South Africa) [31]. This manuscript intends to focus solely on PrEP delivery in the mobile clinic setting. While PrEP delivery at each of these sites follows national guidelines, the POWER study encourages the development and evolution of these delivery models to best fit the contexts of each site and to share ongoing lessons learned.

### The intervention - Cape Town

**Fig. 1:**
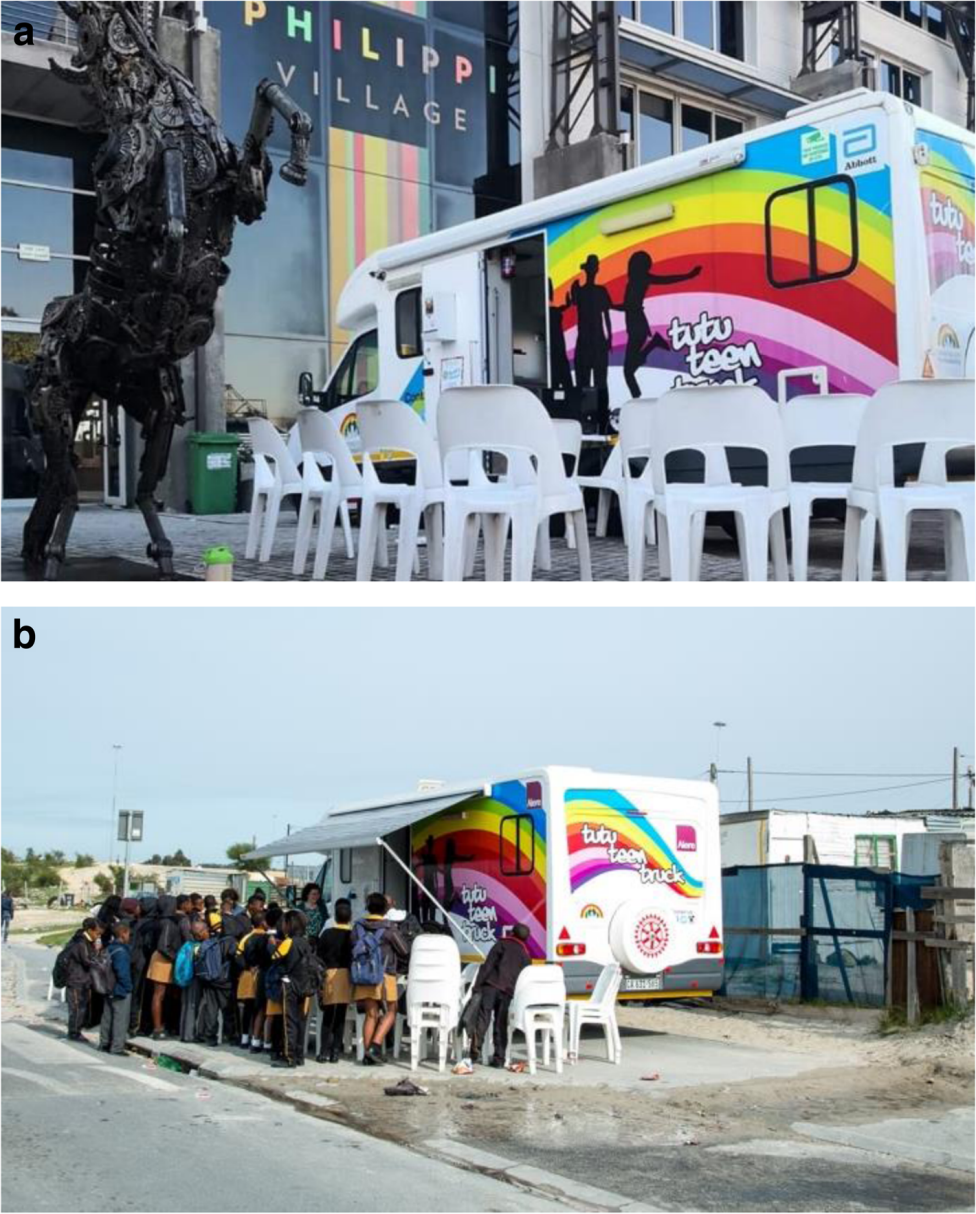
**a** and **b** The Tutu Teen Truck mobile health clinic is a Mercedes Sprinter clinic conversion with private consultation rooms delivering services to adolescent and young people in densely populated, resource limited areas

In Cape Town, the POWER study integrated PrEP delivery into the SRHS provided by the Tutu Teen Truck (TTT) mobile health clinic. The TTT was originally developed in 2015 as an HIV testing and SRHS for adolescents and young people, geographically and logistically distinct from government health facilities’ day-to-day operations. The TTT is a Mercedes Sprinter and trailer clinic conversion with four consultation rooms (see Fig. 1a and b) delivering services in the densely populated, resource limited and high disease burden areas of Cape Town, South Africa.

The TTT aims to increase healthcare access of particularly vulnerable populations by providing services in locations and at times that build on where young women organically network, such as schools, community centers, and public transport hubs. Private, confidential, and non-judgmental adolescent friendly services are provided on the TTT by a clinical nurse practitioner, a professional nurse, and four lay health counselors. Services are tailored to the specific sexual and reproductive health needs of AGYW and include HIV testing, STI testing and treatment, and a range of contraception options (oral, injectable and implant). The TTT utilizes a m-health system (the Broccoli Biometric Profile and Benefits Management System) in which a young woman’s medical record is linked to her fingerprint allowing for anonymized, current, and readily available medical information. A previous study of the TTT indicated that it is a highly acceptable SRHS for adolescents and young adults and preferred over primary health clinics [30].

**Fig. 2:**
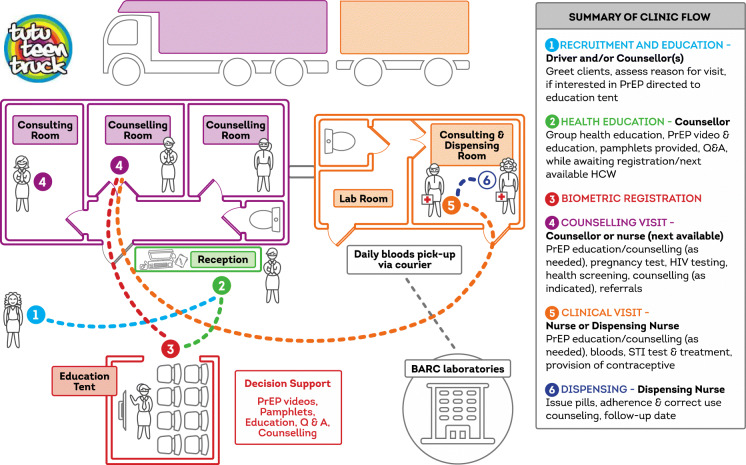
Clinic flow on the TTT mobile clinic include an educational area; four individual consultation rooms for point-of-care HIV and pregnancy testing and health counselling; and a PrEP and contraception dispensing room. Blood and urine samples to verify eligible creatinine clearance and current STI infection (gonorrhoea/chlamydia) are collected by the nurse on the mobile clinic and sent off daily via a courier pick-up service to laboratories for analysis

PrEP delivery was integrated into the TTT sexual health services in July 2017 in consultation with the youth-CAB (community advisory board) to situate the specific intervention within communities largely naïve to PrEP as a biomedical HIV prevention method. AGYW self-presenting at the mobile clinic were invited to view an educational video^1^ about HIV prevalence and risks in their community, and the effectiveness of PrEP for HIV prevention. During individual SRHS consultations, all HIV-negative AGYW were offered PrEP with the option to accept, delay, or refuse uptake. Point-of-care tests for HIV and pregnancy are conducted at every visit before PrEP is dispensed. Blood and urine samples are collected for verification of eligible creatinine clearance (at baseline and 6 monthly), Hepatitis B status (baseline only), and current STI infection (gonorrhea/chlamydia – baseline and 6 monthly) and sent off daily to BARC/Lancet laboratories for analysis via a courier pick-up service (see Fig. 2). Follow-up visits are scheduled 1 month after PrEP initiation and then quarterly, with phone calls and WhatsApp communication for occasional visit reminders. The TTT rotation schedule between its delivery sites is intended to be fixed so that AGYW know when and where they can access follow-up services. The study team attended bi-monthly learning and troubleshooting meetings to ensure the mobile clinic PrEP delivery operations and counseling messaging were adapted to the needs of AGYW within the specific communities of operation.

### Data collection and analysis

For this qualitative sub-study, we purposively selected and telephonically recruited 30 young women (53 were approached) from the PrEP demonstration cohort for in-depth interviews. RTI international applied quota sampling, established through pharmacy records, to include participants with early or continued PrEP use or who discontinued PrEP [32]. The thematic guide was developed by social scientists at RTI international (provided as Supplementary File 1), pilot tested at the study site, and translated into local languages. Interviews and discussions were conducted in participants’ preferred language (English or IsiXhosa) by experienced female social science interviewers (SSI) independent of the demonstration study’s clinical team. All research interviewers had university-level education and were fluent in English and isiXhosa. Young women were advised about the procedures and focus of the research and written informed consent was attained prior to any data collection. IDI’s were conducted one-on-one, in a private community-based venue, lasting approximately 30-45 min and were audio-recorded and transcribed verbatim with sections pertaining to service delivery (clinic setting and logistics) extracted for thematic content analysis (Table 1). Interviews were conducted until saturation of themes was achieved. In addition, notes from six community meetings organized with AGYW receiving PrEP from the TTT and notes from field and study team meetings were included for triangulation analysis.

**Table 1:** Questions from qualitative guides

General Theme	Sample questions/prompts from qualitative guide
PrEP Decision-making	How did AGYW hear about PrEP in their community and/or what encouraged them to come to the clinic and start PrEP?
Clinic Experiences	AGYW asked to describe their clinic experiences, including accessibility, clinic procedures, counseling and support received from staff, and facilitators and barriers to starting and refilling PrEP from the clinic.
PrEP in the future	AGYW were asked about PrEP delivery strategies they think could be helpful for AGYW who want to take PrEP, including convenient locations and times of services and recommendations to clinic experience, PrEP counseling and information.

Relevant information pertaining to key themes of AGYW’s experiences of accessing PrEP from a CMHC, and the benefits and challenges of PrEP delivery in this context was organized by 2 reviewers into major thematic themes using Dedoose^2^ software. Coding was partially deductive drawing from themes in the interview guide but also inductive with emerging themes added to the code book and applied to all transcripts. Throughout the analysis process, samples of the coded transcripts were reviewed by the coding team with any disagreements resolved through discussion until consensus was reached. Study visit medical and pharmacy records collected during young women’s PrEP clinic visits were used to assess participants’ PrEP uptake and patterns of use, and results from this analysis will be described in future studies.

Data collection was approved by the Human Research Ethics Committees of University of Cape Town (593/2016) and University of Washington IRB (00000950) and included a parental waiver of consent for participants younger than 18 years. The POWER study is ongoing; we report here early findings about CMHC delivery of PrEP for this population of AGYW.

## Results

The mobile clinic’s medical records indicate that a total of 1784 AGYW accessed SRHS between July 2017 – October 2019 and 585 (median age of 20 years) initiated PrEP on the same-day. Baseline evidence suggests that the intervention reached the target population who were able to adequately assess their HIV vulnerability and self-select for PrEP initiation. For example, medical records of the AGYW who initiated PrEP indicated 80 % (465) did not know their partner’s HIV status; 85 % (498) were unable to use condoms consistently; and 48 % (259) tested positive for an STI (gonorrhea and/or chlamydia).

The 30 young women participating in the in-depth qualitative interviews had a median age of 20 (18–22) years. Table 2 presents the qualitative participants characteristics. Most AGYW were single with a partner and living with their parents.

**Table 2:** Demographic and behavioural characteristics of participants

	*n* (%)
Age	
16–19	17 (56.7)
20–25	13 (43.3)
Relationship status	
Single, with partner	28 (93.4)
Single, no partner	1 (3.3)
Married	1 (3.3)
AGYW lives with^a^	
Parents	24 (80)
Other family	6 (20)
Sex partner	1 (3.3)
Friends	1 (3.3)
Other	1 (3.3)

^a^AGYW could mark more than one category (‘all that apply’)

Qualitative analysis highlighted five main themes describing the benefits of the CMHC intervention for PrEP delivery: acceptability and accessibility; trusting provider-community-client relationship; non-fragmented and integrated PrEP and SRHS; organic demand generation through AGYW network; and strengthened self-efficacy. A sixth theme was identified highlighting challenges of providing PrEP to AGYW via a CMHC model.

### Acceptability and accessibility of mobile clinic PrEP delivery

Overall, AGYW described how being able to access services integrated in their community and daily lives made the service feel like a place of wellness and protection, rather than diseased-focused. The convenient community locations and high visibility of the TTT encouraged AGYW to access SRHS including PrEP, while eliminating logistical barriers such as transport or difficulties in making appointments. Young women expressed that this made them feel like the service was focused on them and their health needs. Generally, the accessibility, visibility and convenience of the TTT made the services highly acceptable to AGYW and facilitated their uptake of SRHS including PrEP as described here:

*“I used to see this truck regularly; it has been coming to [township name]. I have noticed it while even passing by [in] a taxi. There was this other time, with my friend we thought we should go and test. So when we got there we found out that they [the TTT] also offer family planning, you can test your blood pressure and many more. So we were interested and we tested and got PrEP.”*

*“It’s easy because we get out of school then [the TTT] it’s just next to the school.”*

Young women shared that because the TTT operates at times convenient for them later in the day and the visits are short, it gives them time to still do their homework and chores. Young women valued the convenience and accessibility of the service:

*“When we arrived there [at the TTT], we did not wait long time. They [clinic staff] attended to us immediately in a proper manner.”*

*“You get there [at the TTT] and they [clinic staff] serve you for example if you are there for family planning, after [receiving] that you leave. The other thing that makes it easy is that it [the TTT] is closer to us. We don’t use transport when we are going to the truck… There are no queues [at the TTT], sometimes there will be a lot of people but they [clinic staff] try by all means to be quick.”*

Furthermore, AGYW reported that the high visibility of the TTT and because it is “on their route” frequently acted as a reminder that they are due for a follow-up visit and could do it at that time of remembering without needing an appointment.

### Trusting provider-community-client relationship

The TTT’s community- and patient-centric design facilitated connection and trust between potential clients and health providers. AGYW indicated that services at the TTT are seen as focused on young people making them feel comfortable asking questions about PrEP and sexual health while experiencing non-judgment and privacy. Coming from a community where discussions about sex are often a taboo, they welcomed having a reproductive health conversation with a trusted provider:

*“As he [counsellor] was talking to me, it was as if I was a friend, he was sharing with me in an easily understandable manner. So, I asked lots of questions and he answered.”*

*“I feel I have found someone that can advise me about things that no one can talk to me about.”*

*“And when they [clinic staff] answer me they do it in a manner that I do not feel ashamed.”*

*“I was very comfortable because I am aware that whatever I have discussed with them [clinic staff] will not be discussed with other people.”*

The biometric medical record system allowed for privacy as well as readily accessible client information, overcoming challenges with missing files or difficulty with re-entry when a client has not accessed services recently. Young women also related this feature to a trusting healthcare relationship, indicating their belief that the TTT could be trusted as a consistent service provider for AGYW’s SRHS needs, with no concern about essential product stockouts like at their local primary healthcare clinics. One young woman emphasized her confidence in the reliability of TTT services, *“you will get from the TTT what you came there for*”. Another young woman described her ease of picking up PrEP when she came to an unscheduled visit and conveyed her confidence that she would be able to receive her PrEP refill with ease as a result of the available electronic medical records:

*“It was easy because they [clinic staff] don’t make things difficult, they give you PrEP.”*

### Non-fragmented and integrated PrEP and SRHS

Integrated SRHS offered at the TTT encouraged AGYW to access a holistic prevention approach to their sexual health, with the majority of young women indicating that they came to the TTT for contraception but then also started on PrEP the same day, or vice versa. Overall, young women highlighted the practicality of being able to access a range of services AGYW might need, including HIV testing, STI testing and treatment, PrEP, and contraception.

Furthermore, waiting times at the TTT are integrated with education allowing AGYW to be informed of their health choices by the time they reach the clinical staff.

*“I know the staff there [at the TTT], and so I do not feel like its long [waiting times]. Because when I am there, I enjoy being there and, like, am always talking to people. And the staff is always explaining something new to us…[or] are showing us like the condom stuff and like there is this big book about contraceptives.”*

Providing AGYW with educational information about HIV prevention, including PrEP, and contraception upfront assisted in triaging clients according to their PrEP and SRHS needs and ensures that they follow the most direct route of clinic flow within one clinic visit. A young woman shared her satisfaction with the convenience of integrated SRHS at the TTT:

*“It was good because when you finish with one thing you enter another door immediately.”*

### Organic demand generation through AGYW networks

In the neighborhoods where the TTT operates, AGYW often attend their healthcare visits at the TTT as a group with friends. The TTT is seen as a place that is acceptable to go to with your friends and as being adolescent friendly.

*“It’s nice to be there [at the TTT]. What makes it nice is that when we visit we go as a group with friends. And there is also music. We will stand and listen, you see.”*

The convenient location of the TTT to naturally occurring networks of AGYW, organically facilitated demand generation through young women already on PrEP disclosing their use to their peers and encouraging PrEP uptake. One young woman reported:

*“[W]hen I arrived [at the TTT] there were some children who came to get PrEP and I was not aware [of PrEP] then. I asked these girls if this PrEP is a good thing to do. They said, ‘yes, it is a good thing.’ One was coming for the second time and the other for the third time, they all said they have not experienced any difficulties.”*

Both AGYW and PrEP providers on the TTT reported young women frequently identified peers as influencing their decisions to start PrEP. This experience of AGYW already on PrEP inviting their friends, sisters, and cousins to visit the TTT caused a clear snowball effect in PrEP uptake on the TTT.

### Enabling self-efficacy

AGYW indicated that the trusting relationship they fostered with the TTT along with the education in the waiting area and tailored services they received also fostered self-confidence and motivation to adopt increased health-seeking behavior. AGYW appreciated being informed adequately and in an adolescent friendly and respectful manner and felt it promoted a sense of health ownership and that they have the ability to decide for themselves whether PrEP is an option for them.

*“It is that they [clinic staff] do not get tired when you continue asking them questions. They keep on explaining until you understand, they explain in a way that you easily understand.”*

*“[T]hey [clinic staff] will do tests and tell you about PrEP [and] how it works. Then they will ask you if you would like to get it or not, the choice is entirely yours.”*

Being informed and allowing AGYW to make choices about their health behavior in a manner that fits into their lives leads many young women to confidently merge their contraception and PrEP visit schedules making their SRH visits even more convenient for them.

### Model challenge: mobility of services and young women

A sixth theme was identified highlighting challenges of providing PrEP to young women via a mobile health clinic model. The high mobility of the TTT, the lack of predictability in AGYW’s lives, and the communities in which the TTT operates sometimes led to service delivery interruptions, negatively impacting AGYW’s ongoing access to all SRHS including PrEP via the TTT. Healthcare providers shared that being part of a community means that the TTT experiences the same challenges that members of a community face. In the case of the TTT these included frequent political uprisings, violence hotspots and sometimes severe weather conditions preventing either the TTT or its clients from reaching service sites. While in general the TTT was viewed as highly accessible, some young women shared how the clinic’s mobility caused unplanned PrEP use interruptions:

*“I am still using it, but I have 2 weeks since I stopped taking the pills, because I couldn’t get the truck on the date of the appointment.”*

*“If only it [the TTT] can be bigger …. We usually wait outside. Let’s say it’s raining; we can’t sit outside and we also can’t fit in their rooms so we will be forced to leave.”*

AGYW typically experience limited autonomy in their movement; they mainly moved between school and home and generally did not have the financial resources or social freedom to travel to the TTT when it was outside of their neighbourhood. As a result, a young woman might have started PrEP when the TTT was outside her school but then when away during school holidays, it was difficult for her to obtain PrEP refills as a participant described here:

*“[Sometimes] I do not have money to visit the truck. Or when my [PrEP refill visit] date is not close while they [TTT] visit our school, because I stay far from my school.”*

AGYW’s feedback about how they would like to receive PrEP services in the future frequently included suggestions to mitigate these access barriers, such as expanding delivery locations to include community PrEP clubs, inside their schools, community libraries, community care workers bringing PrEP to their homes, or having a courier drop their refills wherever they are when in need of PrEP.

*“I was thinking, how about getting PrEP delivered door-to-door because sometimes when our [follow-up visit] dates are due we might not be around. We would be maybe travel[ing] to the other province for things like funerals, so when you need it, it would be nice if it can be delivered to your home when not around.”*

*“I thought of asking someone [clinic staff] I know from the mobile truck, if [we] have forgotten our dates they [the TTT] should have a place where we can go and collect our pills.”*

## Discussion

This is the first model of mobile, community-based delivery of PrEP integrated with contraception and other SRHS that has demonstrated feasibility and high acceptability among South African AGYW. CMHC in public health settings are known to increase healthcare access and improve health outcomes in target, yet hard-to-reach, populations such as AGYW in the HIV epidemic [19]. Across our participants’ descriptions of PrEP uptake, the location of the CMHC intervention near where AGYW move or gather was highlighted as very important to their being able to access PrEP services. Beyond the convenience and material advantages of reducing transportation costs, our participants described how having the CMHC in their own community fostered trust, ownership, and positive health seeking behavior. This finding was similarly highlighted in a review of mobile health clinics in the United States, which found that location in surroundings familiar to the community fostered trust [19]. Scholars have postulated that by taking healthcare out of the hospital or doctor’s office and bringing care to the people, the mobile health clinic acts as a vehicle for inclusion, creating empowered spaces for patients to become invested in their healthcare [33].

In addition to the convenience of PrEP uptake from a mobile clinic, AGYW emphasized the importance of services being delivered in an adolescent friendly manner. Their descriptions of what they appreciated about the services covered many of the characteristics embedded in the World Health Organization (WHO)’s recommendation for adolescent friendly services, including treating young people with respect, being non-judgmental and considerate of adolescents needs, and providing appropriate and acceptable services accessible to young people at dedicated service delivery points [34]. Research studies have indicated that further important components of these interventions are anonymized testing, flexible clinic hours, and centering services around AGYW’s significant SRHS needs including contraception and STI treatment [7, 11, 34]. This integration of PrEP and contraception services was of particular significance to young women. The TTT catered for the young women’s comprehensive SRHS needs by providing point-of-care STI testing and treatment and minimizing the number of clinic visits AGYW need to make by integrating PrEP and contraception visits. Notably, while CMHC’s are sometimes criticized for providing fragmented prevention services referring clients to primary health care clinics for additional care [19], the TTT was complimented for providing a non-fragmented service allowing for same-day PrEP start by using a courier lab service and an onboard pharmacy.

Although there are several advantages to the CMHC model for PrEP delivery, it is not an absolute solution. While the mobile clinic’s seamless integration into the community facilitated the acceptability of this PrEP delivery model for AGYW, the CMHC was also negatively impacted by the challenges faced by the broader community, such as community riots, violence and severe weather conditions. During these times, the TTT could not maintain its scheduled visits and return to a predictable schedule and routes was sometimes delayed causing gaps in SRHS delivery including PrEP supply to AGYW. In addition to the disruptions in the delivery described above, AGYW in disadvantaged communities tend to be highly mobile, moving to changing households with the school calendar or as their family or relationship circumstances change.

Mobile PrEP delivery via the TTT facilitated increased uptake of PrEP in AGYW, however additional strategies are needed to support PrEP use continuation in young women. A differentiated model with various delivery options including CMHCs might meet AGYW’s mobility and needs and mitigate supply interruptions which occur through reliance solely on the CMHC. The Tutu Teen Truck recently implemented a differentiated PrEP delivery model allowing AGYW to choose between four modes of PrEP delivery: (1) community-based mobile health clinic; (2) local government clinic; (3) community-based PrEP club; and (4) courier PrEP delivery service. These differentiated delivery models will need to pay special attention to overcome linkage/continuity challenges that have proven challenging for HIV and TB treatment from CMHC to static clinic sites [29, 35–37] and perhaps apply lessons learned from successfully community-based ART distribution programs [38]. Future implementation research should move beyond scale-up strategies for PrEP to also include scale-out strategies where an established PrEP delivery intervention is rolled out to novel settings to enhance AGYW’s uptake and persistence on PrEP [39].

The results presented in this paper are preliminary while the study is still ongoing in this community and cohort. Caution is needed with regard to generalizing our findings beyond AGYW in limited resource settings in South Africa, as attitudes and experiences across communities and groups might differ from this limited sample.

## Conclusions

PrEP delivery from a mobile health clinic is feasible and acceptable to AGYW in South Africa. The Tutu Teen Truck facilitated a gender-responsive approach to delivering PrEP by providing a comprehensive service to AGYW’s reproductive health needs, bringing the services close to young women taking into account their limited autonomy, and meaningfully involving AGYW in the planning of service uptake and continuation strategies. Additionally, positive provider-client interactions and trustworthy service delivery was combined in the model to strengthen self-efficacy and health-seeking behavior among AGYW. However, findings also highlighted the importance of differentiated service delivery beyond CMHC as PrEP is scaled-up across South Africa. Diverse PrEP delivery locations and modalities will need to be developed so as to fit into the complex lives, mobility, and competing demands of AGYW and adapted to navigate contexts of chronic social instability in South Africa.

## Supplementary Information

**Additional file 1.** Thematic Guide: IDI with Young Women

## Data Availability

The de-identified dataset/transcripts used and/or analysed during the current study are available from the corresponding author on reasonable request.

## Abbreviations

AGYW: Adolescent Girls and Young Women
ART: Anti-retroviral Treatment
CAB: Community Advisory Board
CMHC: Community Mobile Health Clinic
HIV: Human Immunodeficiency Virus
POWER: Prevention Options for Women Evaluation Research
PrEP: Pre-Exposure Prophylaxis
SRHS: Sexual Reproductive Health Services
STI: Sexually Transmitted Infections
TB: Tuberculosis
TTT: Tutu Teen Truck
WHO: World Health Organisation

## Footnotes

1 https://www.youtube.com/watch?v=c-vDZMzuLvY

2 Version 6.1.18, Los Angeles, CA: SocioCultural Research Consultants, LLC www.dedoose.com
