# Supplementary material for: A community-based mobile clinic model delivering PrEP for HIV prevention to adolescent girls and young women in Cape Town, South Africa
Source: BMC Health Serv Res. 2021 Aug 28;21:888. doi: 10.1186/s12913-021-06920-4 (PMC8403426; doi:10.1186/s12913-021-06920-4)
Supplement: Supplementary file 1 — Additional file 1. Thematic Guide: IDI with Young Women [file 12913_2021_6920_MOESM1_ESM.docx]

Thematic Guide: IDI with Young Women

**INSTRUCTIONS for the Interviewer: How to use the IDI Guide**

1. Section topics are in **bold text**.
2. Scripts to introduce the IDI, sections and questions appear in normal font prior to the section/questions.
3. There are two levels of questions:

- Primary interview questions: are numbered, indicating required questions for the interview. They address the topics that you as the interviewer must ask and discuss with participants. The questions are suggestions for getting the discussion going. You are not required to read them verbatim, but they are written to ensure some consistency across IDIs. You may adapt the questions and/or ask them in a different order, depending on how the discussion develops. However you will have to ensure that by the end of the discussion, all the topics and answers to the primary questions have been covered.
- Probing topics: are lettered (a, b, c…) or use small roman numerals (i, ii, iii…). If you find that the participants provide little information in response to the primary question, these probing topics may be used to encourage further discussion. You are not required to cover every topic listed. So, depending on what has already been discussed, and the IDI context, you may ask these probes or not.

1. *Instructions to interviewer are in* ***bold italics and [brackets]. It is important to read these to know the correct questions to pose to each participant group.***
2. Words found in (parentheses) are meant to provide examples that the interviewer can use to clarify the question if necessary.

Thematic Guide: IDI with Young Women

**Theme 1: Knowledge of PrEP *[For initial acceptors, restarters and refusers]***

1. How did you hear about PrEP at first? What do you know about PrEP?
   1. Where did you hear about or who told you about PrEP?
   2. Did you hear anything about PrEP in the media? (e.g. radio, advertising, social media?)
   3. What have you heard about PrEP?
      1. Who can take PrEP and why?
      2. How is it taken?
2. What interested you about PrEP?
   1. What made you want to learn more about PrEP?
3. What encouraged you to come to the clinic?
   1. Was there anyone who motivated you to come?

**Theme 2: PrEP Decision *[For initial acceptors/refusers, restarters and late acceptors]***

I’d like to ask about your initial decision about PrEP:

1. How did you decide if taking PrEP was right for you or not at the beginning?
   1. What were the pros and cons you considered when making the decision?
   2. How did people in your life influence your decision about whether or not to take PrEP? (e.g. partner, family, or friends)
   3. What health concerns did you consider when making your decision?
      1. Concerns or risk of getting HIV
      2. Concerns from taking PrEP (side effects, long term health effects)
   4. How did the requirements for getting PrEP and taking it regularly influence your decision of whether or not to use it?
2. ***[If this question is not clear, offer examples:]*** Accessing PrEP, time spent at the clinic; privacy; ability/willingness to take daily; forgetting; pill storage, pill burden, HIV testing
3. How did information from clinic staff influence your decision about PrEP?
   1. What did the counselors or the health provider tell you?
   2. Did you talk about any of your concerns about PrEP?

**PrEP decision tool*:*** Let’s discuss a tablet/Ipad program you might have used while you were waiting for your appointment, which we’re calling the “PrEP decision tool”.

1. Did you use the decision tool? ***[If she did not use the tool, skip the rest of this section]***
2. How useful was the tool in helping you make decisions about taking or not taking PrEP? Please describe:
   1. Specifically, what section within the tool helped you decide?
   2. What information from the tool was the most useful to helping you to decide? Why?
3. What can be changed or improved with the tool?
4. The content or sections of the tool itself ***(if participant has trouble remembering tool components, you may remind her of these)***:
   1. 1.0-3.0 **Opening screens** (including “Reasons Why”)
   2. 4.0 **Options**: PrEP, Condoms, Decreasing my sex partners, Knowing if my partner has HIV, If my partner has HIV, he takes HIV medicines
   3. 5.0 **PrEP Versus PEP**
   4. 6.0-11.1 **Let’s talk about PrEP** menu: What is it, Why take PrEP, How well does it prevent HIV, How do I take PrEP, What you need to know about PREP, True and False, etc.
   5. 12.1 **Learn More** – checking off options to talk to the clinic staff about
   6. 13.0 **Family planning**
5. When and where it is used

***[For initial refusers or if she states that didn’t use PrEP]:***

1. Can you imagine a situation in which you may want to use PrEP? If yes, please describe to me.

***[For restarters and late acceptors only]:***

1. What made you change your mind and decide to start using PrEP?
   1. Did you find out any information, including rumors, that made you change your mind about using PrEP? Could you explain?
   2. How did people in your life influence your decision to change your mind about using PrEP? (e.g. partner, family, or friends)
   3. Do you think a change in your ideas about your level of risk influenced your choice to start using PrEP? How?
      1. Risk of getting HIV
      2. Risks from taking PrEP (side effects, long term health concerns)
   4. Did any other change in your life make it easier to take PrEP? (household, work, financial, other) How?
   5. Additional Probes for restarters:

- How long were you off PrEP? How long since you resumed PrEP use?
- Were there any new situations or change in circumstances that gave you motivation to restart?

1. Did anything happen at the clinic that made you decide to start using PrEP? Tell me more.

***[For all participants]:***

**Theme 3: Clinic Experience**

1. Now I would like to talk about your clinic visits. How easy or difficult was it to come to the clinic? What made it easy or difficult?
2. Describe what happens when you go to the clinic for a PrEP visit.
3. How easy or difficult was it to get services and PrEP at the clinic? What made it easy or difficult?
4. If you’ve gotten refills, how was it when you went to get refills? Where did you go?
5. Can you describe counseling and support you received on how to take PrEP?
6. What were you told?
7. What were the things you liked about it?
8. What were the things you disliked about it?
9. Do you feel they addressed your questions and concerns? Could you give me an example?
10. How do you think you were treated during this clinic visit?
    1. Can you give an example of what was good or bad?
    2. Do you feel that you have received any discrimination or judgment from clinic staff? If so, can you give an example?

1. How comfortable were you in discussing topics related to sex, HIV, PrEP and family planning with the clinic staff you interacted with during your POWER visit?
   1. Can you give an example of a time when you felt comfortable or uncomfortable talking about these topics? What makes/can make you feel more at ease?
   2. How comfortable were you asking the study staff questions?

**Theme 4: Experiences using PrEP *[For anyone who took PrEP: initial acceptors, late acceptors, restarters, persistors, non-persistors, and seroconverters]***

Now I would like to talk about your experience using PrEP.

1. How has taking PrEP been for you?
   1. Tell me about the last time you took a pill… when was it, at what time, what were you doing, and where were you?
   2. Where have you been storing the pills? Any challenges with storage?
2. How has taking PrEP made you feel about yourself?
   1. How have you felt physically? (Any side effects? Any positive effects)
   2. How have you felt emotionally?
   3. How did you feel (emotionally/physically) during the PrEP pause?
3. How often do you take PrEP?
4. What are the things that have helped/made it easy for you to take PrEP? Please describe any strategies that you have used to help you take PrEP.
5. What are the things that have made it difficult for you to take PrEP?
   - 1. How have you dealt with these challenges?
6. How well do you think you are protected with the # of doses you’ve taken?
7. Tell me about a time you missed taking your PrEP pill.
8. What caused you to miss doses?
9. Did you talk to the clinic staff about it? If no, why not? If yes, what did they say? Was it helpful?
10. What could help you to prevent missing doses?

***[For restarters, non-persistors or for persistors who reveal gaps in use]***

1. If you stopped using PrEP for any amount of time, or didn’t take pills you picked up, what made you stop?
2. Did you find out any information, including rumors, that made you change your mind about using PrEP? Could you explain?
3. How did people in your life influence your decision to stop using PrEP? (e.g., partner, family, or friends)
4. Were there any changes in your life (in your household, work, income, other) that made it harder to take PrEP?
5. Do you think a change in your ideas about your level of risk influenced your choice to stop using PrEP? How?
   - 1. Risk of getting HIV
     2. Risks from taking PrEP (side effects, long term health concerns)
6. Was taking a pill daily difficult? Please describe your difficulties.
7. Did you use other forms of protection while on the PrEP pause?
8. Was stopping PrEP an active, intentional choice? Or was it due to circumstances out of your control?
9. Are there any other reasons that caused you to stop taking PrEP?
10. Did anything happen at the clinic that made you decide to stop using PrEP? Tell me more.
    1. Did you continue to visit the POWER clinic during your PrEP pause?
    2. Did you have any worries about returning to the clinic after your PrEP pause?
11. Do you think you will use PrEP in the future? Why/Why not?
    1. Are there any barriers or facilitators in your life and personal relationships that act as barriers or facilitators to PrEP use?
    2. After restarting, what are your future goals for PrEP use? How can the clinic team support you?

**Theme 5:** **Relationships**

1. Now let’s talk about your relationships. Are you currently in any relationships? (Main partner, casual partners)
   1. Tell me about your main partner and what your relationship is like.
   2. Do you have other partners?
      1. Tell me about your relationships with them.
   3. In the relationship(s) you’re in, have you ever talked about HIV with your partner(s)?
2. ***[If yes]*** Tell me more about the discussions you’ve had.
3. Have you ever had an HIV test together with your partner(s)?
   1. ***[If yes]*** Do you know your partner(s) HIV test status?
   2. Have you ever experienced any form of violence in your relationship(s)? (Physical, emotional, sexual, psychological)
4. Have you told anyone else that you are using PrEP or that you were thinking about using PrEP? Why/why not?
5. ***[If yes]*** Who have you told? (partner, friends, family) And how did that go?
6. Can you tell me about how people in your life have reacted to your use of PrEP?
7. Whose reaction has been most important?
8. Who supported you in taking PrEP? How?
9. Who discouraged your use of PrEP? What did they say?
10. Who has supported your decision to restart PrEP? Did anyone oppose it?
11. Is there anyone important that you are hiding it (PrEP) from?
    - - 1. Is there anyone important that you didn’t tell? Why?
12. ***[If no]*** What made you decide to keep your use of PrEP a secret?
13. What kind of support or advice have you received from clinic staff on talking about PrEP to your partner(s)/family?
14. What did they tell you? Did they say how or whether to tell partner/ family?
15. How did they address the challenges you faced?
16. In what ways was the support helpful/adequate?
17. How could the support be improved?
18. What kind of information about PrEP was available for you to give to partner(s)/family?
19. What other resources would you like to give or to share with your partner(s)/family?

***[If she took PrEP at all]:***

1. Has using PrEP affected any of your family relationships? How?
2. Has using PrEP affected your sexual relationships? How? (e.g. decision-making, empowerment, communication)
   1. How about condom use with sexual partners? Has that changed?

**Theme 6: Stigma and Discrimination**

1. Now I would like to talk about what people in the community are saying about PrEP. Can you tell me about any things you have heard in your community about PrEP or this study? ***[This is however she defines community – can be neighborhood, peers, etc]***
   1. What kinds of ideas or rumors have you heard circulating?
   2. Who have you heard these from?
   3. How do you feel about what you’ve heard?
   4. ***[If untrue]*** What do you think is the best way to address these ideas/rumors?
      1. Who should address these?
      2. Is there anything the clinic has done or could do to address community rumors or stigma?

**[If she used PrEP]:**

1. Can you tell me about any positive or negative social experiences you have had for using PrEP?
   1. What do they say?
   2. Who says those things? Tell more about these types of people. (Partner, family, friends, community, health care workers)
   3. How do you feel about what they say?
      1. Do you think what they say is true or false? Why?

**Theme 7: Seroconversion** *[****For seroconverters only]***

If it’s okay with you, I’d like to talk about your HIV status.

1. What in your view might have contributed to you getting HIV?
   1. Can you tell me more about any particular time or event when you think you may have gotten HIV?
   2. Before getting infected, did you experience any changes in your personal life, behaviors, or circumstances that could have increased your chances of getting HIV?
      1. Changes in PrEP adherence
      2. Changes in other prevention behaviors
      3. Changes in relationships
   3. How do you feel being in POWER impacted your risk of getting HIV?
   4. How do you feel being on PrEP could have influenced your chances of getting HIV?
2. What support did you receive from the clinic staff when you got your HIV test results?
3. Was it helpful? If yes, how? If not, how could it improve?
4. Tell me about your experiences with accessing HIV care.
5. Have you been referred to an HIV care clinic?
6. Have you been to an HIV care clinic yet?
   - 1. ***[If yes]*** How did the PrEP clinic staff support you in going there?
     2. Are you currently on ART?
     3. ***[If yes]*** How was your experience with starting ART?

**Theme 8: Advice** *[****For Persistors, Non-Persistors, restarters, and Seroconverters who have been in the study for 3 months or more****]*

Now I would like to learn more about what you have said, or would say, to other young women about PrEP.

1. Have you ever advised other young women about using PrEP?
   1. ***[If yes]*** Who have you talked to? In what venues or spaces? (e.g. at school, on social media, in community groups)
   2. ***[If no]*** Why not?
2. What kind of advice have you given to other young woman about PrEP? ***[Interviewer: If the participant has never given advice, ask her to imagine what she might say if she were asked.]***
3. Should she take it?
4. What should she know?
5. How should she address any challenges that may come up using PrEP?
6. Would you advise her to tell other people (partner, family, friends) about her PrEP use?
7. What kind of changes in her life should she expect if she uses PrEP?
8. What kind of advice would you give her about using condoms while using PrEP?

**Theme 9: PrEP in the future** *[****For persistors, non-persistors, restarters, and seroconverters who have been in the study for 3 months or more****]*

Now I want us to talk about what PrEP delivery should look like in the future.

1. Which locations or surroundings would be convenient and safe to go and get PrEP?
   1. How far would you be willing to travel to get PrEP?
   2. How long would a reasonable wait time be for a PrEP clinic appointment be?
   3. What locations and hours would be most convenient to get PrEP?
2. How can the clinic experience be made better?
3. How should the clinic procedures be made better?
4. How should the clinic space or environment be made better?
5. How should the procedures for refills be made better?
6. What kind of provider would you prefer to go to for PrEP prescriptions and refills?
7. Please describe to me your experience where you felt comfortable with a provider ***[To determine the characteristics of providers that she is comfortable with]***
8. What would you change about clinic staff to make your experience more comfortable?
9. How could the clinic staff help young women store PrEP safely and privately at home?
10. What kind of information or counseling do you think could be helpful for young women who want to take PrEP?
11. What kind of social support from partner, friends, family, young women need to help them take PrEP?

***[All participants]***

**Theme 10: As we finish**

1. Do you have any other thoughts or questions? ***[Write down questions and make sure to answer them or refer the participants as needed].***
2. Are there any other questions we should have asked about how to improve delivery of PrEP or ease of use?
